# Diversity and Dynamics of Microbial Community Structure in Different Mangrove, Marine and Freshwater Sediments During Anaerobic Debromination of PBDEs

**DOI:** 10.3389/fmicb.2018.00952

**Published:** 2018-05-15

**Authors:** Ya Fen Wang, Hao Wen Zhu, Ying Wang, Xiang Ling Zhang, Nora Fung Yee Tam

**Affiliations:** ^1^Laboratory of Basin Hydrology and Wetland Eco-restoration, School of Environmental Studies, China University of Geosciences, Wuhan, China; ^2^Department of Biology and Chemistry, City University of Hong Kong, Kowloon Tong, Hong Kong; ^3^School of Civil Engineering and Architecture, Wuhan University of Technology, Wuhan, China; ^4^State Key Laboratory in Marine Pollution, City University of Hong Kong, Kowloon Tong, Hong Kong

**Keywords:** PBDEs, mangrove sediment, microbial community, reductive debromination, T-RFLP, salinity

## Abstract

Little is known about the diversity and succession of indigenous microbial community during debromination of polybrominated diphenyl ethers (PBDEs). This study examined the diversity and dynamics of microbial community structure in eight saline (mangrove and marine) and freshwater sediment microcosms exhibiting different debrominating capabilities for hexa-BDE 153, a common congener in sediments, using terminal restriction fragment length polymorphism (T-RFLP) and clone library analyses. The results showed that microbial community structure greatly differed between the saline and freshwater microcosms, likely leading to distinct variations in their debrominating capabilities and pathways. Higher relative abundances of Chloroflexi and Deltaproteobacteria succeed by Alphaproteobacteria and Betaproteobacteria were detected in the two mangrove microcosms with the fastest debrominating capabilities mainly via *para* pathway, respectively; the dominance of Alphaproteobacteria resulted in less accumulation of tetra-BDEs and more complete debromination of lower brominated congeners (from di- to tetra-BDEs). Meanwhile, the shifts in both microbial community structure and PBDE profiles were relatively small in the less efficient freshwater microcosms, with relatively more *ortho* and *meta* brominated products of BDE-153 resulted. Coincidently, one of the freshwater microcosms showed sudden increases of Chloroflexi and Deltaproteobacteria by the end of incubation, which synchronized with the increase in the removal rate of BDE-153. The significant relationship between microbial community structure and PBDEs was confirmed by redundancy analysis (18.7% of total variance explained, *P* = 0.002). However, the relative abundance of the well-known dechlorinator *Dehalococcoides* showed no clear correlation with the debrominating capability across different microcosms. These findings shed light in the significance of microbial community network in different saline environments on enhancement of PBDE intrinsic debromination.

## Introduction

Polybrominated diphenyl ethers (PBDEs) are highly diverse, toxic and persistent organohalides, prevalent in sediments and soils around the world (Oros et al., 2005; Binelli et al., 2007; Li et al., 2012; Zhu et al., 2014a; McGrath et al., 2017). They share the structural similarity and environmental fate with other highly halogenated compounds like polychlorinated biphenyls (PCBs) and have been included in Stockholm Convention (2009) on the list of persistent organic pollutants (POPs). Concerns about their bioaccumulation and potential endocrine, reproductive and behavioral toxicity effects to human and wildlife have posed urgent needs for their remediation (Lam et al., 2010; Chakraborty and Das, 2016).

Microbial debromination of PBDEs played a major role in PBDE dissipation in soil, compared to plant uptake and other physiochemical processes (Huang et al., 2010). It has been well documented that PBDEs could be transformed to less-brominated congers under both aerobic and anaerobic conditions by bacterial isolates or consortia (Robrock et al., 2008; Lee et al., 2011; Ding et al., 2013; Zhang et al., 2013). The only key biodegradation mechanism for certain recalcitrant and highly brominated BDE congeners is reductive dehalogenation, during which the organohalides could serve as electron acceptors for bacteria to derive energy for their growth under anaerobic conditions (Mohn and Tiedje, 1992). This specific group of bacteria is known as organohalide-respiring bacteria (OHRB), which have been identified from limited bacterial phyla of Chloroflexi, Firmicutes, and Proteobacteria (Hug et al., 2013). Different OHRB exhibited highly specialized capabilities for the debromination of different BDE congeners (He et al., 2006; Robrock et al., 2009; Yang et al., 2015), suggesting that complete or effective remediation of PBDEs required the cooperation of different OHRB, and with other bacterial functional groups such as those capable of cleavage of aromatic rings. Moreover, *in situ* microbial remediation process would largely depend on the degradation network of the indigenous microbial community present (Lovley, 2003). However, our current knowledge of the diversity and dynamics of indigenous microbial community involved in the whole PBDE debrominating process is very limited.

Previous studies of PBDE remediation were mainly conducted in electronic wastes contaminated soils, sewage sludge and river sediments (Yen et al., 2009; Shih et al., 2012; Song et al., 2015; Stiborova et al., 2015). Microbial debromination could be enhanced by addition of co-substrates or electron donors, priming with other halogenated compounds, and stimulation with electrochemical technology (Gerecke et al., 2005; Qiu et al., 2012; Yang et al., 2013; Huang et al., 2014). More recent studies followed the application of new functional materials such as tourmaline, nanoscale zero-valent iron and carbonaceous materials during PBDE remediation (Zhang et al., 2014; Cai et al., 2015; Ma et al., 2016; Zhu et al., 2016). But exploration of PBDE debromination under natural environments were rather limited, especially in the marine/saline environments (Zanaroli et al., 2015). Nevertheless, our previous study showed that different types of sediments varying in salinity had great impacts on debromination of hexa-BDE153. More rapid and complete reductive debromination was observed in mangrove and marine sediments than in freshwater sediments (Zhu et al., 2014b). Although microbial reductive dechlorination potential and activities have been reported in marine coastal and subseafloor sediments (Futagami et al., 2013; Matturro et al., 2016a), studies focusing on the role of indigenous microbial community in reductive debromination in marine environments is scarce.

Additionally, most of the known OHRB were isolated from freshwater environments, with only seven (out of 74) from marine and estuarine sediments, and the phylogeny and functional diversity of certain genus like *Dehalococcoidia* were found more likely regional specific, e.g., greatly differing between marine and freshwater environments (Atashgahi et al., 2016; Zinder, 2016). Thus, the distribution of the extensively studied *Dehalococcoidia* in marine environments and their role in intrinsic debromination are also of particular interest to be explored.

In the present study, the structure and dynamics of indigenous microbial community were examined across eight different sediment microcosms varying in reductive debrominating capabilities for BDE-153 over a course of 90 days. We aimed to test the hypothesis if the debromination potential of different sediments was determined by their indigenous microbial community structure. Besides, the phylogeny of the microbial community involved in PBDE debromination was revealed to infer the role of the known OHRB during intrinsic debromination.

## Materials and Methods

### Sample Description

Sediment slurry samples were taken from the batch microcosm experiment for evaluation of the intrinsic debromination potential of different sediments collected from Hong Kong, SAR. There were eight sediment microcosms, including five mangrove sediments from Mai Po Ramsar wetland (MP), Sha Tau Kok (STK), Ting Kok (TK), Ho Chung (HC), and Tai O (TO), two freshwater pond sediments from Mai Po (MPf) and Nam Sang Wai (NSW), and one marine sediment from Sai Kung (SK) (Supplementary Figure S1). The five intertidal mangrove sediments (MP, STK, HC, TK, and TO) were dominated by *Kandelia obovata*, with the salinity ranged from 29 to 35‰, pH from 7.55 to 7.92 and the total organic matter (TOM) from 2.07 to 8.98%, while the two freshwater sediments (MPf and NSW) were dominated by *Phragmites australis* with the salinity from 6 to 8‰, pH from 7.35 to 7.48 and the TOM from 6.59 to 7.22%. The marine sediment (SK) had a salinity of 34‰, pH of 8.08 and TOM of 3.54%.

The detailed set-up of the microcosms has been described by Zhu et al. (2014b). In brief, the minimal salt medium (MSM) containing (in g L^-1^): K_2_HPO_4_, 0.27; KH_2_PO_4_, 0.35; NH_4_Cl, 2.7; MgCl_2_6H_2_O, 0.1; CaCl_2_2H_2_O, 0.1; and the trace elements made up of FeCl_2_4H_2_O, 0.009; MnCl_2_4H_2_O, 0.004; ZnCl_2_, 0.0014; CoCl_4_6H_2_O, 0.001 and (NH_4_)_6_ Mo_7_O_24_4H_2_O, 0.001 was prepared as described previously (Li et al., 2009). The salinity was adjusted to be the same as that in the original sediment sample by adding appropriate amounts of NaCl into the MSM. The medium was autoclaved at 121°C for 30 min, then freshly prepared L-cysteine and sodium sulfide (Na_2_S) (0.2 mM each) were added aseptically to eliminate residual oxygen. For each microcosm, 100 mL of the sterilized MSM were dispersed into a 250-mL Quickfit conical flask, 20 g of fresh sediment were then aseptically added in an anaerobic glove box under the flux of nitrogen gas and mixed thoroughly with the medium to form the sediment slurry. The anaerobic condition was maintained using N_2_ refilling method, and all the microcosms were acclimated for 2 weeks. After acclimation, 200 μL stock solution of BDE-153 (Chem Service, AccuStandard, purity >99%) at 100 mg L^-1^ in acetone was spiked into each microcosm to obtain a nominal concentration of BDE-153 at 1.0 mg kg^-1^ (freeze-dried weight). The sediment slurry was shaken after spiking to let the slurry equilibrated with BDE-153 and let the small volume of acetone (0.2%) evaporated according to the previous study of Zhong et al. (2006). The actual spiked concentration of BDE-153 at day 0 was determined to be 1.08 ± 0.08 mg kg^-1^ (mean and standard deviation of three replicates). The solvent control with acetone was not included, as Fagervold et al. (2005) reported that the solvent control without the target pollutants, i.e., PCBs, showed little effects on the microbial dechlorinating activity and community profiles. All the microcosms in triplicates were incubated at 28°C on a horizontal shaker at 150 rpm for 90 days, to ensure the homogeneity of the sediment slurry. At regular time intervals, a 2 mL slurry was subsampled from each microcosm using sterile disposable syringes for PBDE analysis, in parallel, another 2 mL slurry was collected at days 1, 15, 30, 60, and 90, and stored at -70°C for later microbial community analysis.

### DNA Extraction and PCR Amplification

Genomic DNA was extracted from 0.25 g of sediment using the FastDNA^TM^ SPIN Kit for Soil (MP Biomedicals, Irvine, CA, United States). The bacterial 16S rDNA genes were amplified using the universal primers 8F: 5′-AGA GTT TGA TCC TGG CTC AG-3′ and 1492R: 5′-GGC TAC CTT GTT ACG ACT T-3′ (Lane, 1991). The 8F primer was labeled at the 5′ end with 6-carboxy-fluorescein phosphoramidite (FAM). The PCR cocktail composed of 10 μL 5× colorless GoTaq^TM^ Flexi Buffer, 3 μL of MgCl_2_ (25 mM), 1 μL of dNTP (10 mM), 0.5 μL of each primer (10 μM), 0.25 μL of GoTaq^TM^ Hot Start Polymerase (5 U μL^-1^) (Promega, Madison, WI, United States), 1 μL template DNA (3–10 ng), and the volume was made up to 50 μL using nuclease-free H_2_O. The PCR condition was an initial denaturation at 94°C for 3 min; 30 cycles of 94°C, 45 s denaturation; 55°C, 45 s annealing; 72°C, 60 s extension; followed by a final extension at 72°C for 7 min. The PCR products (1.5 kb) were then verified by running a 1.2% (w/v) agarose gel electrophoresis in 1× Tris-acetate-EDTA (TAE) buffer (pH 7.4).

### Terminal Restriction Fragment Length Polymorphism (T-RFLP) Analysis

The PCR products were concentrated and purified using the AxyPrep^TM^ DNA Gel Extraction Kit (Axygen, United States), which resulted in sufficient abundance of T-RFs for detection after digestion with the restriction enzyme (the next step). Aliquots of the purified PCR products (500–800 ng) were digested with the restriction enzyme *Hae*III at 37°C according to the manufacture’s protocol (New England Biolabs, Inc., United Kingdom). The total volume of the digestion system was 40 μL. Digested products were precipitated by two volumes of cold absolute ethanol at -20°C overnight, centrifuged at 14,000 rpm at 4°C for 20 min and washed with 100 μL of 70% cold ethanol. After centrifugation, the DNA pellets were dissolved in 20 μL of nuclease-free water. Purified products (10 μL) were mixed with 0.5 μL of the internal size standard (ET ROX-550, Amersham, United States). This mixture was denatured for 2 min at 95°C and immediately chilled on ice before electrophoresis on a MegaBASE genetic analyzer (Amersham, United States) operated in a Genotyping mode at the Coastal Marine Laboratory, Hong Kong University of Science and Technology.

The basic T-RF calling was performed using a Genetic Profiler in the MEGABACE software package (Amersham, United States). For each sample, peaks over a threshold of 50 fluorescence units were used, and T-RFs of <60 bp and >550 bp were excluded from the analysis to avoid detection of primers and uncertainties of size determination, respectively. The raw data file exported from MEGABACE was re-formatted and then processed by T-REX software developed by Culman et al. (2009), which helped to determine a baseline threshold for the identification of true peaks over noise, align T-RFs in all samples and construct data matrices based on the area of T-RF peaks.

A phylogenetic assignment tool, TRiFLe program for *in silico* T-RFLP analysis with user-defined sequence sets, was adopted (Junier et al., 2008). A total of 2,533 bacterial sequences tagged with “marine sediments” were selected from the RDP database and used as the reference data set for the simulation of the T-RF size distribution and identification of the candidate species in the sediment samples.

### Cloning and Phylogenetic Analysis

16S rRNA gene fragments from the genomic DNA from microcosms were amplified with the universal bacterial primer pairs of 8F and 1492R as described above. The PCR amplicons were checked by agarose gel electrophoresis, purified and cloned to pGEM-T Easy Vector System I (Promega, Madison, WI, United States) according to the manufacturer’s instructions. Positive colonies were selected randomly and sent to a company (Beijing Genomics Institute, Shenzhen, China) for sequencing. Near-complete 16S rRNA gene sequences were obtained and compared to other sequences via the Basic Local Alignment Search Tool (BLAST) of the National Centre for Biotechnology Information (NCBI^1^). The 16S rRNA gene sequences obtained from the clone libraries in this study have been deposited in NCBI GenBank database under the following accession numbers: MH091074- MH091326. The phylogenetic tree was constructed by the neighbor-joining method in MEGA 5.0 package as described by Tamura et al. (2011).

### Quantification Analysis of *Dehalococcoides* sp. by qPCR

Real-time PCR was performed using an ABI 7300 real-time PCR instrument (Applied Biosystems, Carlsbad, CA, United States) with genus-specific primers for *Dehalococcoides* sp. (Deh467F and Deh564R) and universal primers for total bacteria (341F and 534R) (Muyzer et al., 1993; Freeborn et al., 2005). The amplification was performed in 25-μL reaction mixtures composed of 1×ABI SYBR^®^ Green Master Mix (Applied Biosystems, Carlsbad, CA, United States), 0.2 μM of each primer and 2 μL of DNA template, containing 1–10 ng μL^-1^ of DNA. The amplification program and efficiency has been described in our previous study (Wang et al., 2015). The relative abundance of *Dehalococcoides* sp. was determined by normalizing the quantitative results of the specific genes to the total amount of bacterial 16S rRNA gene copies within the same sample (van der Zaan et al., 2010).

### Statistical Analysis

The peak area data from T-RFLP analysis were analyzed by clustering and multidimensional scaling (MDS) analyses, as suggested by Culman et al. (2008), for the T-RFLP datasets with high beta diversities (≥2). Clustering and MDS analyses for the matrix of peak area were based on [Log(x+1)] transformation of percentage values and Bray–Curtis similarity. Significant differences between neighboring samples were evaluated by similarity profile (SIMPROF) tests with a significance level at 0.05 in cluster analysis. Exploratory tool similarity percentage analysis (SIMPER) was also used to study the similarity between complex T-RFLP profiles. The above analyses were performed using the PRIMER 6 software package (Clarke and Gorley, 2006). The PAST program (PAlaeontological STatistics ver.1.64) was used to calculate the Shannon and Simpson diversity indices. The relationship between the profiles of T-RFLP and different PBDE congeners were investigated using redundancy analysis (RDA), after checking the length of gradient of T-RFLP dataset (to be 3.360) by detrended correspondence analysis (DCA) in the CANOCO program for Windows v4.5 from Microcomputer Power (Ithaca, NY, United States). Monte Carlo permutation test was performed to test the significance of the first and all canonical axes in RDA.

## Results

### Debrominating Pattern of BDE-153 in Different Sediments

**FIGURE 1:**
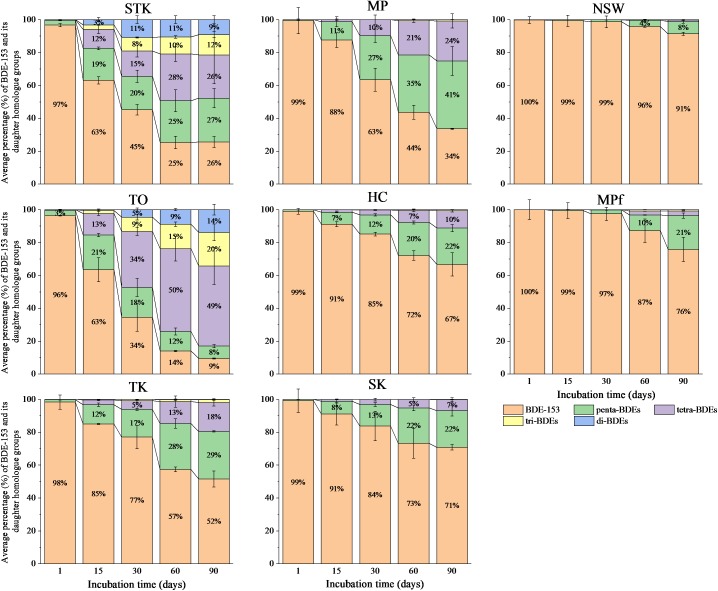
Debromination pattern of BDE-153 to less-brominated congeners over time in the eight sediment microcosms. Data plotted are the mol percentage of the peak area of a BDE congener to the total peak areas of all BDE congeners identified in GC/MS chromatograms. Mean values of three replicates are shown. STK, TO, TK, HC, and MP are intertidal mangrove sediments collected from Sha Tau Kok, Tai O, Ting Kok, Ho Chung and Mai Po, respectively; SK is marine sediment collected from Sai Kung; MPf and NSW are fresh pond sediments collected from Mai Po nature reserve and Nam Sang Wai.

Our previous study showed that reductive debromination of BDE-153 was detected more extensive in mangrove and marine sediments (the removal rate of BDE-153 up to approximately 98% after 90 days), than in the freshwater pond sediments. The half-lives of BDE-153 in the sediment microcosms varied from 7.6 days in STK to 165.0 days in MPf (Zhu et al., 2014b). The sequential debrominating products of hexa-BDE 153 ranging from penta- to di-BDEs were found in all the sediment microcosms after 90 days, however, the dominant BDE homolog groups varied greatly among sediments with different debrominating rates (**Figure 1**). Lower brominated congeners (di- and tri-) were detected within 15 days in STK and TO, the two most efficient sediment microcosms, and their proportions increased after 30 days of incubation, accounted for around 20% in STK and 14–34% in TO, while BDE-153 rapidly decreased during this period. In contrast, the proportions of these lower brominated congeners in the other sediment microcosms were very low (less than 3%). Penta- and tetra-BDEs were found as the main daughter BDE congeners in STK and TO, accounting for 53–62% of the total BDE congeners. However, tetra-BDEs were more dominant than penta-BDEs in TO after 30 days of incubation but the percentages of tetra- and penta-BDEs were more or less the same in STK, suggesting TO had a more rapid transformation of BDE-153 and penta-BDEs but slower transformation of tetra- to lower brominated products, which was quite different from that in STK. In the other mangrove and marine sediment microcosms, penta- and tetra-BDEs formed in a sequential manner, and their respective proportions ranged from 7 to 41% and from less than 3 to 24%. Only 4 to 21% of penta-BDEs were found in the freshwater microcosms (NSW and MPf) after 60 days onwards, and tetra-BDEs were even lower (less than 3%), suggesting much lower transformation abilities for hexa-BDE 153 in the freshwater microcosms.

### T-RFLP Analysis of Total Microbial Communities

**FIGURE 2:**
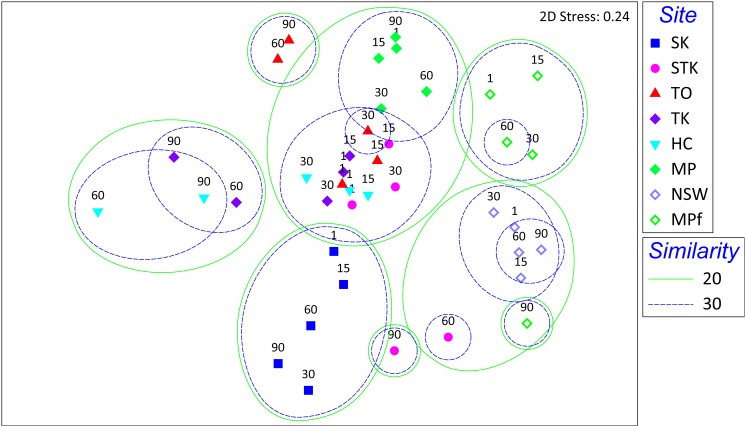
Sample scatter plot of multidimensional scaling (MDS) analysis of T-RFLP profiles of total bacterial community from the eight sediment microcosms at Days 1, 15, 30, 60, and 90 after spiked with BDE-153. Neighboring samples clustered at a similarity level of 20 and 30% are indicated.

The 16S rRNA fragment genes were fingerprinted with T-RFLP to monitor the dynamics of microbial community structure across 40 different sediment samples during debromination of BDE-153. Totally, there were 270 T-RFs generated by *Hae*III digestion, with an average richness (number of T-RFs in each sample) of 36.1. The sample heterogeneity (also known as beta diversity) of T-RFLP dataset was very high (to be 6.5, greater than 5) as suggested by McCune and Grace (2002). The heterogeneity of the microbial community from replicated samples of the same treatment was small according to our previous study using fatty acid methyl ester (FAME) analysis and others with T-RFLP analysis (Sipilä et al., 2008; Wang Y.F. et al., 2014). Non-linear ordination method, MDS was thus employed to resolve the similarity of our T-RFLP data, which included 40 sediment samples at five sampling times from Days 1 to 90(**Figure 2**).

All the sediment samples were firstly clustered along the salinity gradient, with mangrove and marine sediments on the left side and freshwater sediments on the right side of the MDS scatter plot. The mangrove sediments could be further separated with marine sediments from SK along the second axis, except for STK sediments collected after Days 60 and 90. The average similarity for each sediment site was calculated by SIMPER analysis, showing the temporal change was greatest in STK (21.94) and minimized in NSW (39.32). From the scatter plot, all the mangrove sites showed a clear temporal shift after Days 60 and 90 during the debrominating process, as well as for the freshwater sediment microcosm MPf after 90 days. By contrast, such temporal shifts were insignificant in marine microcosm SK and another freshwater microcosm NSW at a similarity level of 30%.

**FIGURE 3:**
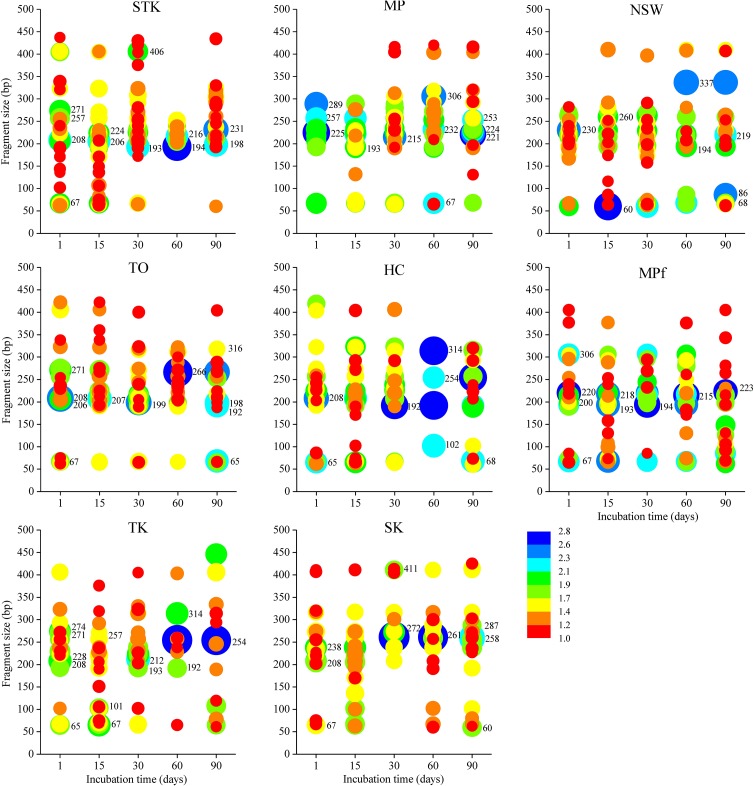
Relative abundance of *Hae*III digested 16S rDNA T-RFs from total bacterial communities in the eight sediment microcosms from Days 1 to 90. The symbol color and size indicate the relative abundance of each T-RF as percentage of total peak area (log transformed).

Dynamics of the dominant terminal restriction fragments (T-RFs) were examined to identify the key bacterial species involved in PBDE debromination (**Figure 3**). For the two most active mangrove microcosms STK and TO, they shared T-RFs 206, 271 and 208 at Days 1 and 15, while the number of their shared T-RFs decreased from 23 to 9 after 90 days, with T-RFs 216, 231, and 194 dominated at STK and T-RFs 266 and 316 dominated at TO after Day 60, respectively. T-RFs 271 and 208 were also detected as dominant T-RFs in TK and HC at Day 1, but the dominant T-RFs shifted to 254, 314, and 192 bp in TK and HC after 60 days. Accordingly, the T-RFLP patterns in TK and HC were the most similar (average dissimilarity = 74.20, SIMPER) compared with other pairs of sediment microcosms. The dominant T-RFs in marine sediment SK resembled those detected in mangrove sediments, except for T-RFs 238(at D1–D15), 261 (at D30–D90) and 411. The freshwater sediment NSW also held unique dominant T-RFs such as 230 (at D1–D15) and 337 (at D60–D90). However, it was interesting to find that both mangrove and freshwater sediments collected from MP shared certain dominant T-RFs, such as 193, 215, 221–225, and 306, but these T-RFs became dominant at different timing.

Molecular identification of the dominant T-RFs with cumulative contribution to the differences in T-RFLP profiles over time greater than 50% in each sediment microcosm were summarized (**Table 1**). The dominant T-RFs in mangrove and marine microcosms from Days 1 to 30 were identified to contain 16S rRNA gene sequences similar to uncultured deltaproteobacterium clones (T-RFs 206, 208, 238) and two strains from the Firmicutes family of *Clostridiaceae* (T-RFs 257 and 271). The two T-RFs 225 and 220 were abundant in mangrove and freshwater microcosms from Mai Po at Day 1 and both belonged to the phylum of Chloroflexi. The partial 16S rRNA sequences of dominant T-RFs from Days 60 to 90 matched with more bacterial phyla, including Actinobacteria (T-RF 231, STK), Betaproteobacteria (T-RF 198, STK), Alphaproteobacteria (T-RFs 192, 253/254, mangrove microcosms), candidate division OP8 (T-RF 261, SK), and Bacteroidetes (T-RF 411, SK). T-RFs 266 and 316 enriched in TO after Day 60 were closely related to *Clostridium algidicarnis* and another uncultured Firmicutes, respectively. T-RF 215 shared by the two microcosms from Mai Po after Day 30 were 98% similar in the partial 16S rRNA sequence to one uncultured deltaproteobacterium clone, which was also detected from the anaerobic microbial community in oil-polluted subtidal sediments reported by Acosta-González et al. (2013).

**Table 1:** Molecular identification of the dominant terminal restriction fragments (T-RFs) with cumulative contribution to differences of T-RFLP profiles over time greater than 50% in each sediment microcosm by SIMER analysis.

Predicted T-RF length (bp)	Observed T-RF length (bp)	Phylogenetic group	Dominant in	Closest match from the clone libraries constructed in this study	
**Dominant at Day 1 ∼ Day 30**				
68	67	Gammaproteobacteria	All	Uncultured gamma proteobacterium (AM259852.1), 99%	
208	208	Deltaproteobacteria	Saline^a^	Uncultured delta proteobacterium (EF061950.1), 98%	
206	206	Deltaproteobacteria	STK, TO	Uncultured delta proteobacterium (DQ395025.1), 97%	
237	238	Deltaproteobacteria	SK	Uncultured delta proteobacterium (EF655671.1), 96%	
257	257	Firmicutes; Clostridia	Saline	Uncultured Clostridiaceae bacterium (FJ425601.1), 96%	
270	271/272	Firmicutes; Clostridia	STK, TO, TK, SK	*Alkaliphilus peptidofermentans* Z-7036 (EF382660.1), 91%	
224	225/224	Chloroflexi; Anaerolineae Anaerolineae	MP	Uncultured *Anaerolineaceae* bacterium (JF806903.1), 97%	
221	220	Chloroflexi	MPf	Uncultured Chloroflexi bacterium (FM242343.1), 95%	
**Dominant at Day 60 ∼ Day 90**					
231	231	Actinobacteria	STK	Uncultured *Actinobacteridae* bacterium (FN582328.1), 98%	
195	193	Gammaproteobacteria	STK	Uncultured gamma proteobacterium (JF344548.1), 96%	
196	194	Deltaproteobacteria	MPf, NSW	*Desulfovibrio* sp. enrichment culture clone (HQ108123.1)^b^	
266	266	Firmicutes; Clostridia	TO	*Clostridium algidicarnis* NCFB 2931 (X77676), 95%	
199	198	Betaproteobacteria	STK, TO	Uncultured beta proteobacterium (AM713401.1), 100%	
316	314/316	Firmicutes	TO, TK, HC	Uncultured Firmicutes bacterium (AM745203.1), 94%	
192	192	Alphaproteobacteria	TO, TK, HC	*Pseudoruegeria aquimaris* (NR_043932.1), 92%	
254	253/254	Alphaproteobacteria	TK, HC	Uncultured alpha proteobacterium (FJ666151.1), 90%	
260	261	Candidate division OP8	SK	Uncultured candidate division OP8 (DQ811949.1), 97%	
414	411	Bacteroidetes	SK	Uncultured Bacteroidetes bacterium (JF344262.1), 95%	
310	306	Firmicutes; Bacilli	MP, MPf	*Bacillus* sp. G11001 (AB531397.1), 99%	
215	215	Deltaproteobacteria	MP, MPf	Uncultured delta proteobacterium (JF344345.1), 98%	

*^a^Saline sediments refer to all mangrove and marine sediments. ^b^The underlined closest match is from the reference species rather than from the clone libraries in this study*.

### Microbial Diversity and Phylogeny Involved in Debromination

**FIGURE 4:**
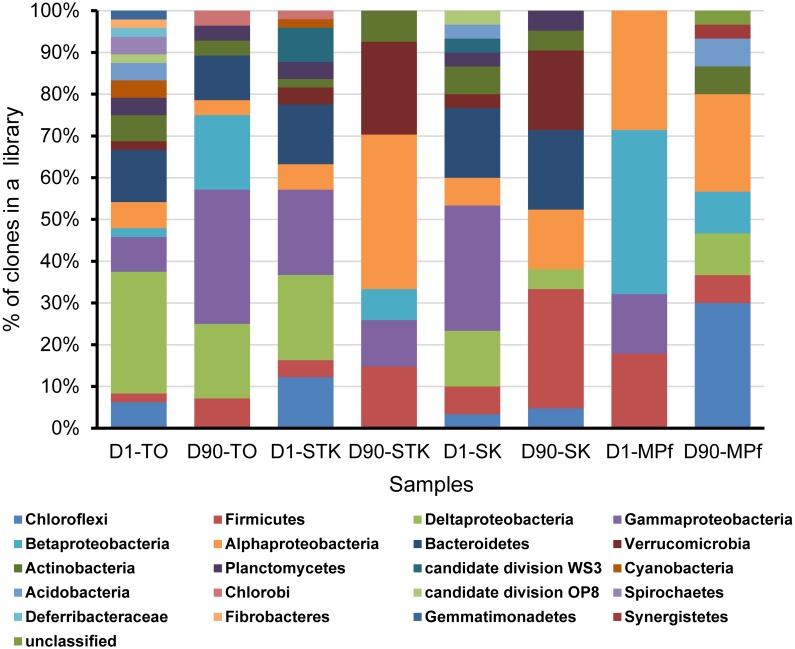
Phylogenetic composition of the eight 16S rRNA gene clone libraries constructed from STK, TO, SK, and MPf sediment microcosms at Days 1 and 90, respectively.

Eight bacterial 16S rRNA clone libraries were constructed from four representative anaerobic microcosms of TO, STK, SK, and MPf at Days 1 and 90, respectively. Phylogenetic analysis revealed that 261 full-length 16S rRNA sequences were related to 20 bacterial phyla, including Gamma-, Alpha-, Delta-, and Beta-proteobacteria (*n* = 130, 50%), Bacteroidetes (*n* = 28, 11%), Firmicutes (*n* = 27, 10%), and Chloroflexi (*n* = 20, 8%). Other phyla with a few clones (less than 5% of the total), such as Verrucomicrobia, Actinobacteria, Planctomycetes, Acidobacteria, Gemmatimonadetes, and Chlorobi, as well as two candidate phyla WS3 and OP8, were also present (**Figure 4**). The phylum-level diversity distinctly decreased in the mangrove and marine microcosms (TO, STK, and SK) from 2.3 at Days 1 to 1.8 at Day 90 in average, while that in the freshwater microcosms (MPf) increased from 1.3 to 1.9. The distribution of dominant phyla also significantly differed between the saline and freshwater microcosms over time (**Figure 4**). The clones within Gamma-, Delta-proteobacteria, Bacteroidetes, Chloroflexi, and Firmicutes divisions from the saline microcosm libraries were more abundant than those from the freshwater ones, with distribution percentages accounting for 90, 90, 100, 55, and 74% of the total detected in the saline libraries, respectively. Furthermore, the percentages of the clones from the above first four phyla in the saline libraries detected at Days 1 and 90 dropped from 61 to 29%, 83 to 7%, 71 to 29%, and 50 to 5%, respectively. The respective values of the Alpha-, Beta-proteobacteria, and Firmicutes clones increased from 19 to 38%, 5 to 32%, and 22 to 52% in the saline libraries, but decreased from 24 to 19%, 50 to 14%, and 19 to 7% in the freshwater ones. The bloom of Chloroflexi (from 0 to 45%) was observed in MPf at Day 90, followed by that of Deltaproteobacteria (from 0 to 10%). Cluster analysis, in good accordance with the T-RFLP profiles, revealed a close clustering of the samples from MPf at Day 90 with those from STK and SK at Day 90, although the samples from STK, TO and SK microcosms differed greatly from MPf at Day 1 (Supplementary Figure S2).

**FIGURE 5:**
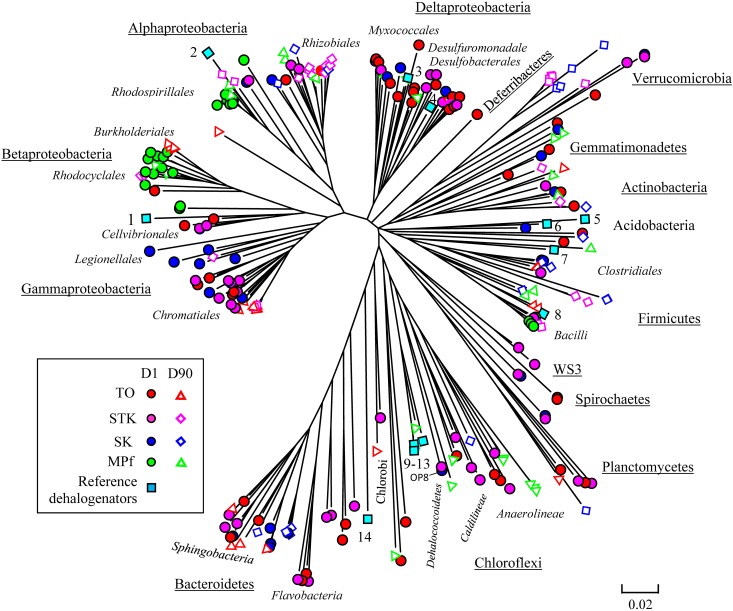
Neighbor-joining phylogenetic tree of the 261 16S rRNA nucleotide sequences obtained from the STK, TO, SK, and MPf sediment microcosms at Days 1 and 90 with reference to members of 14 known dehalogenators (1) *Shewanella sediminis* strainHAW-EB3 (NR_074819.1); (2) *Ruegeria* sp. TM1040 (CP000377.1:144962-146416); (3) *Anaeromyxobacter dehalogenans* strain 2CP-1 (AF382396.1); (4) *Desulfomonile limimaris* (AF282177.1); 5. *Desulfovibrio* sp. TBP-1 (AF090830.1); (6) *Desulfovibrio dechloracetivorans* strain SF3 (NR_025078.1); (7) *Acetobacterium* sp. AG (JQ627627.1); (8) *Dehalobacter restrictus* (U84497.2); (9) *Dehalobium chlorocoercia* DF-1 (AF393781.1); (10) *Dehalococcoides* sp. BAV1 (AY165308.1); (11) *Dehalococcoides mccartyi* GY50 (NC_022964.1:c841590-840096); (12) *Dehalococcoides* sp. MB (EU073964.1); (13) *Dehalococcoides mccartyi* strain 195 (NR_114415.1); and (14) *Dehalospirillum multivorans* (X82931.1).

A phylogenetic tree of all the 16S rRNA gene clones was constructed to clearly display the phylogenetic distribution of the whole microbial community across different microcosms and their phylogenetic association with 14 known dehalogenators (**Figure 5**). It was further explored on the major differences in the microbial community structure between STK and TO at Day 90, as these two most efficient microcosms also showed distinct PBDE profiles. *Chromatiales*, *Rhodocyclales*, and *Clostridia* were ranked as the three most abundant bacterial classes in TO, while *Verrucomicrobia, Rhizobiales* and unclassified *Alphaproteobacteria* were the three most dominant classes in STK (**Table 2**).

**Table 2:** Relative abundance (%) of classes of Proteobacteria, Chloroflexi, and Firmicutes that known to contain organohalides-respiring bacteria (OHRB).

(Sub) Phylum	Class	TO		STK		SK		MPf		Representative species in the clone library
		D1	D90	D1	D90	D1	D90	D1	D90
Alphaproteobacteria	Rhizobiales	2.1	0	2.1	18.5	0	10	0	6.7	Uncultured *Hyphomicrobium* sp.
	Rhodobacterales	0	0	4.3	7.4	3.0	0	31.0	10	*Thioclava* sp.
	Rhodospirillales	2.1	3.7	0	0	3.0	5.0	0	0	Uncultured *Rhodovibrio* sp.
	Sphingomonadales	0	0	0	0	0	0	0	6.7	Uncultured Sphingomonadales bacterium
	Unclassified-alpha	0	0	0	11.1	0	0	0	0
Betaproteobacteria	Burkholderiales	0	0	0	7.4	0	0	17.2	10	*Ralstonia* sp.
	Rhodocyclales	2.1	18.5	0	0	0	0	17.2	0	*Denitromonas indolicum*
	Nitrosomonadales	0	0	0	0	0	0	3.4	0	*Thiobacillus thioparus*
Deltaproteobacteria	^∗^Desulfobacterales	10.4	7.4	8.5	0	3.0	0	0	3.3	Uncultured Desulfobacteraceae bacterium
	^∗^Desulfuromonadales	6.3	0	2.1	0	0	0	0	3.3	Uncultured *Desulfuromonas* sp.
	^∗^Myxococcales	4.2	0	0	0	3.0	0	0	3.3	Uncultured Myxococcales bacterium
	Syntrophobacterales	2.1	0	2.1	0	3.0	0	0	0	Uncultured Syntrophaceae bacterium
	Unclassified-delta	2.1	0	4.3	0	3.0	0	0	0
Gammaproteobacteria	Chromatiales	2.1	25.9	6.4	7.4	12.1	0	13.8	0	Ectothiorhodospiraceae bacterium
	Cellvibrionales	2.1	0	4.3	0	3.0	0	0	0	*Haliea* sp.
	Legionellales	0	0	2.1	3.7	3.0	0	0	0	*Legionella* sp.
	Thiohalophilus	0	3.7	0	0	3.0	0	0	0	*Thiohalophilus thiocyanatoxydans*
	Thiotrichales	0	0	0	0	3.0	0	0	0	Candidatus *Thiopilula aggregata*
	sulfur-oxidizing symbionts	0	3.7	0	0	0	0	0	0
	Unclassified-gamma	8.3	0	6.4	0	6.1	0	0	0
Chloroflexi	Anaerolineae	4.2	0	0	0	3.0	0	0	10	Uncultured Anaerolineaceae bacterium
	Caldilineae	0	0	0	0	0	0	0	6.7	Uncultured *Caldilinea* sp.
	^∗^Dehalococcoidetes	0	0	0	0	0	0	0	3.3	*Dehalogenimonas lykanthroporepellens*
	Sphaerobacteridae	0	0	0	0	0	0	0	3.3	*Sphaerobacter thermophilus*
	Unclassified-Chloroflexi	2.1	0	12.8	0	0	5.0	0	6.7
Firmicutes	Bacillales	0	0	2.1	7.4	3.0	0	17.2	0	*Bacillus* sp.
	^∗^Clostridia	0	11.1	2.1	7.4	6.1	35.0	0	0	Clostridiaceae bacterium
	Unclassified-Firmicutes	2.1	0	0	0	0	0	0	6.7
Overall relative abundance of the entire bacterial community		52.1	74.1	59.6	70.4	60.6	55.0	100	80

*^∗^The class harboring the pure isolates of the known OHRB (Atashgahi et al., 2016). ^1^http://www.ncbi.nlm.nih.gov*

Of the 20 OTUs of Chloroflexi, only one OTU (D90-MPf-22), identified as *Dehalogenimonas lykanthroporepellens* (95% similarity), fell into the class of *Dehalococcoidetes* and closely clustered with the most extensively studied *Dehalococcoides* spp. (Bootstrap value = 87). Another OTU of two Firmicutes clones (D90-TO-14 and D90-TO-22) were identified as *Desulfitobacterium* sp. and affiliated with *Dehalobacter restrictus* (U84497.2) (Bootstrap value = 100). One OTU (D90-STK-4) identified as *Ruegeria* sp. were also clustered with the Alphaproteobacterial OHRB *Ruegeria* sp. TM1040 (CP000377.1:144962-146416) (Bootstrap value = 64).

### Molecular Detection and Quantification of *Dehalococcoides* 16S rRNA Genes

All the sediment microcosms held 16S rRNA gene copies of the total bacteria and *Dehalococcoides* within the range of 10^6^–10^9^ and 10^2^–10^6^, respectively, suggesting the relative abundance of *Dehalococcoides* sp. within the whole microbial community was less than 1%. The relative abundances of *Dehalococcoides* sp. were above 10^-4^ at Day 1 in all the sediment microcosms, followed by “down-up” fluctuations from Days 1 to 30, and the fluctuations varied at different degrees among sediment microcosms in the later stage (Supplementary Figure S3). Notably, the relative abundances of *Dehalococcoides* sp. were found much higher in NSW at each sampling time (around 10^-4^ ∼ 10^-3^) but became the lowest in the marine sediment SK after 60 days (as low as 10^-6^).

## Discussion

### Relationship Between Microbial Community Structure and Their Debrominating Capability

**FIGURE 6:**
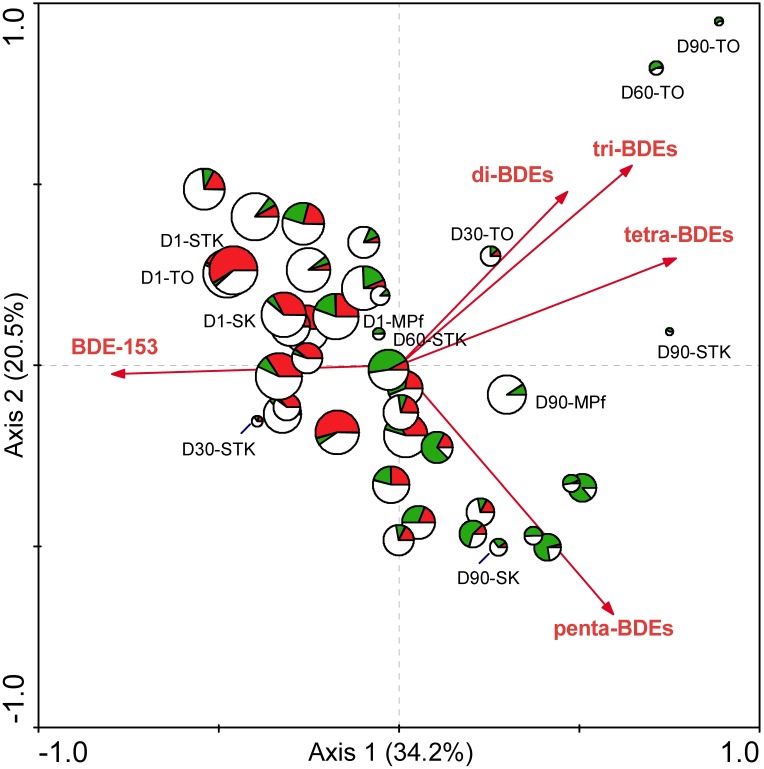
The biplot diagram with PBDEs and samples based on RDA analysis of the T-RFLP and PBDE profiles from the eight sediment microcosms from Days 1 to 90, with the size of the sample symbols corresponding to total PBDE concentration, and the pies of the sample symbols corresponding to the distribution of different groups of T-RFs that were defined in **Table 1**. (red: dominant T-RFs at Days 1 ∼ 30; green: dominant T-RFs at Days 60 ∼ 90; blank: the remaining T-RFs detected).

To our knowledge, this study presents the first attempt to compare the microbial community structure from different types of sediments with varying reductive debrominating capabilities. This is a crucial step to explore the key debrominating microorganisms responsible for the *in situ* bioremediation processes (Lovley, 2003). The distribution of the well-known OHRB, i.e., *Dehalococcoides*, *Dehalobacter*, and *Desulfitobacterium*, has previously been examined across debromination microcosms with 28 different soils and sediments, but their presence or absence was not necessarily related to the debrominating activity of samples (Lee and He, 2010). In the present study, the distinct different debrominating capabilities of sediment microcosms between saline and freshwater environments have been shown to rightly correspond to the differences in their microbial community structures (**Figure 2**). The significant relationship between microbial community structure determined by T-RFLP profiles and PBDE debrominating pattern was further confirmed using RDA, as previously demonstrated by Xu et al. (2012)). The first and all canonical axes contributed to explain 6.4 and 18.7% of the total variance in microbial species data, with *F*-values of 2.321 (*P* = 0.005) and 1. 563 (*P* = 0.002), respectively. The biplot with PBDEs and samples displayed clearly that the large variance of T-RFLP profiles among different samples was mainly attributed to the debromination degree in terms of total PBDE concentration and types of BDE congeners (**Figure 6**). This result complemented our previous study (Zhu et al., 2014b), and provided valuable evidence into the role of indigenous microbial community in the fate of PBDEs during natural attenuation. In addition, it extended our knowledge regarding PBDE debrominating capability mainly derived from studies with isolated dehalogenating bacteria or enriched cultures (He et al., 2006; Robrock et al., 2009; Zeng et al., 2010), which supported the differences in microbial community structure could lead to different debrominating capabilities, owing to the substrate specificity and specific debrominating pathways adopted by different OHRB species present.

The clone library analysis revealed that the mangrove and marine microcosms exhibiting more rapid and extensive debromination of PBDEs harbored a more diverse microbial community than that in the freshwater microcosm at Day 1. The positive correlation between bacterial diversity and their degradation efficiency has also been reported by Dell’Anno et al. (2012). More importantly, the distinct distribution patterns of the dominant bacterial phyla were observed between the saline and freshwater microcosms. Deltaproteobacteria and Chloroflexi were more abundant in the saline microcosms, while Alpha- and Beta-proteobacteria dominated the freshwater microcosm at the 1st day. According to the phylogenetic distribution of the known OHRB, Deltaproteobacteria and Chloroflexi are the two major phyla that harbor non-obligate and obligate OHRB, respectively (Atashgahi et al., 2016). The Chloroflexi strains, including *Dehalococcoides* and *o*-17/DF-1 like bacteria, have frequently been identified as the key dehalogenators in the chlorinated organics-contaminated sediments, but the complete removal of the substrates usually took a few months or years (Zanaroli et al., 2012; Wang and He, 2013). However, one study reported that the co-culture consisting of *Dehalococcoides* and *Desulfovibrio* (Deltaproteobacteria) strains exhibited a rapid and complete debromination of tetra- and penta-BDEs within 14 days (Lee et al., 2011). These findings could partially explain the rapid and extensive debrominating capabilities of microbial communities from STK and TO microcosms in the present study.

Meanwhile, Alpha- and Beta-proteobacteria that dominated in MPf at Day 1 became one of the dominant phyla in STK and TO microcosms, respectively, at Day 90, when they exhibited distinctly different PBDE profiles (**Figures 1**, **4**). The dominance of Alphaproteobacteria has also been documented in PCB dechlorinating microcosms of marine sediments by the later stage of incubation (Matturro et al., 2016c). The cooperation between Alphaproteobacterial members with the potential PBDE debrominating strains from Deltaproteobacteria and Chloroflexi was inferred by their succeed dominance in different stages of PBDE debromination, and suggested to contribute to more rapid and complete debromination in STK microcosms compared to in TO microcosms that dominated by Beta- and Gamma-proteobacteria at the same time (**Table 2**). The Alpha- and Beta-proteobacterial members were reported to be capable of degrading less recalcitrant and lower brominated BDE congeners (from mono- to hexa-BDEs), such as *Sphingomonas* sp. PH-07, *Rhodococcus jostii* RHA1 and *Burkholderia xenovorans* LB400, owing to their aromatic ring-cleavage activity (Kim et al., 2007; Robrock et al., 2009). However, information on the reductive debromination of Alpha- and Beta-proteobacteria was rather limited. It is thus essential to gain deep insights in their metabolic interactions with the known OHRB for the complete debromination of PBDEs in contaminated sediments.

Unique distribution of *Planctomycetes*, *Gemmatimonadetes*, and *Verrucomicrobia* in the saline sediments were detected. These phyla have also been revealed to be actively involved in organic carbon utilization in the deep-sea environments (Li et al., 2016). Besides, most of the key T-RFs enriched were identified to belong to the known OHRB phyla, except for T-RF 261 being assigned to candidate division OP8. In our previous study on constructed mangrove microcosms for treatment of a mixture of wastewater-borne PAHs and PBDEs, a candidate division OP8 clone showing a high affinity with the *Dehalococcoides mccartyi* 195 was also detected (Wang et al., 2015). It was also once reported in a hydrocarbon- and chlorinated-solvent contaminated aquifer during intrinsic remediation (Dojka et al., 1998) and as representative members in mangrove sediments (Ikenaga et al., 2010). Further investigation on the role of these phyla in reductive debromination is needed and may help to discover new OHRB from marine/saline environments.

### Dynamics of Microbial Community Structure and Their Debrominating Capability

It was found that the more rapid and divergent succession of microbial communities in the saline microcosms largely coincided with the changes in PBDE profiles. In contrast, the shifts in both microbial community structure and PBDE profiles were relatively small in the freshwater microcosms (**Figures 1**, **2**). Interestingly, a sudden change in the MPf microcosm till Day 90 was detected by T-RFLP analysis, with Chloroflexi and Deltaproteobacteria greatly enriched, and the removal rate of BDE-153 also greatly increased as observed in the mangrove and marine microcosms after Day 15. These results suggested that the microbial debrominating capability was not only determined by the microbial community structure, but greatly associated with the magnitude and dynamics of microbial community shifts over time. In fact, the succession of microbial community structure has been well documented in response to oil spill and could be used for prediction of the on-going remediation progress (Kimes et al., 2014). However, much less is known about microbial community shifts in response to PBDEs, further investigation is required to provide direct evidences, such as stable-isotope probing (SIP) and functional gene data, on the contribution of the enriched functional bacterial groups to PBDE debromination.

Relic DNA (referred to large amounts of extracellular DNA or in non-intact cells) has been demonstrated to significantly influence the structure of the microbial community, and its effects depended on the pH value of soil samples, which ranged from 3.5 to 8.0 as reported by Carini et al. (2016). Although the interference from relic DNA was not determined in the present study, the interference could be reflected by the dynamic analysis of the microbial community structure in each sediment microcosm, which just meant to resolve the succession of microbial communities. The pH values of the eight sediment samples in our study were all slight alkaline, from 7.4 to 8.0, so the effect of pH on relic DNA interference was considered insignificant as the pH gradient was relatively narrow. In addition, the presence of relic DNA did not obscure the detection of distinct shifts and sediment type-specific patterns in microbial communities from our sediment microcosms varying in the debromination capability.

### The Role of Known OHRB During Intrinsic Debromination

So far only one isolate from the Chloroflexi, *Dehalococcoides mccartyi* GY50 could couple its growth with debromination of PBDEs and lead to more rapid and complete debromination compared to the co-metabolic pattern adopted by previously reported debrominating cultures (Ding et al., 2017). Detection and quantification of the well-known OHRB in dehalogenation microcosms have been widely applied to evaluate their role in intrinsic remediation processes (van der Zaan et al., 2010). Here, we only quantified 16S rRNA genes of *Dehalococcoides* because of the following reasons. Most of the known OHRB were isolated from the freshwater environments, with only seven originating from marine and estuarine sediments. Among the seven marine-originated OHRB isolates, two strains, i.e., *o*-17/DF-1 like bacteria, belong to the genus of *Dehalococcoides*, while the other five belonging to Delta- and Gamma-proteobacteria were all non-obligate OHRB, according to Atashgahi et al. (2016). We focused on monitoring the abundance of obligate OHRB, as many obligate degraders were identified as the key members responsible for the rapid bioremediation in marine environments, e.g., after oil spills (Yakimov et al., 2007). However, the obligate degraders were suggested as a minor seed population that would quickly withdraw after relief of the pollution stress (Yang et al., 2016). In the present study, *Dehalococcoides* were not dominant in the microbial community, and their relative abundances varied during the debromination process, with no clear pattern observed in relation to their debrominating efficiency across different microcosms (Supplementary Figure S1). For the role of OHRB in intrinsic debromination, Xu et al. (2012) found that that the known dechlorinating bacteria, such as *Dehalobacter*, showed low abundances in the enriched PBDE-degrading communities and some unclassified members could be involved in debromination by using barcoded pyrosequencing. In another recent study on tetrachloroethene (PCE) contaminated marine sediments, it has been found that the relative abundance of non-*Dehalococcoides* Chloroflexi retrieved from the original sediment took the predominance over *Dehalococcoides* sp. that added after 150 days, suggesting the important potential role of non-*Dehalococcoides* Chloroflexi during natural reductive dechlorination process (Matturro et al., 2016b). These results suggested that one or more unknown highly efficient debrominators rather than *Dehalococcoides*-like species would be present to perform extensive debromination in our microcosms.

Furthermore, different OHRB strains from even the same genus as *Dehalococcoides* showed quite different dehalogenation capabilities due to the different number and types of reductive dehalogenases (RDases) genes they possess (Hug et al., 2013). Recently, novel PCB dechlorinase genes *pcb*A1, *pcb*A4, and *pcb*A5 have been revealed in *D. mccartyi* strains by Wang S. et al. (2014), together with three PBDE RDases genes *pbr*A1, *pbr*A2, and *pbr*A3 in *D. mccartyi* strain GY50 by Ding et al. (2017). These important discoveries would provide more specific biomarkers targeting reductive dehalogenase genes for evaluation the role of the known OHRB in debromination processes in more depth.

## Conclusion

This is the first study comparing the microbial community structure and dynamics across different sediments during intrinsic debromination of PBDEs. Both the microbial community structure and their succession largely attributed to variations in the debrominating capabilities and pathways among sediment types. Mangrove and marine sediment microcosms with relatively higher abundance of Chloroflexi and Delta-proteobacteria showed faster debromination compared with freshwater microcosms. Moreover, nearly complete debromination of BDE-153 could be achieved with Alpha-proteobacteria being enriched in the later stage. Freshwater microcosms exhibited relatively slow debromination but the regeneration of those functional debrominating microorganisms were detected by the end of incubation. Further investigation is needed to compare the functional diversity of microbial communities involved in debromination in saline and freshwater sediments, or under different salinity gradient, combined with culture-dependent approaches and powerful deep sequencing and environmental microbiome technologies. These findings would improve our understanding on the role of indigenous microbial community in the debromination of PBDEs in natural sediments, and indicate the great potential to enrich and isolate debrominating microorganisms from mangrove sediments for further bioremediation purposes.

## Author Contributions

YW and NT designed the study. YW, HZ, and YW performed the microbial and PBDE analyses. All authors wrote the manuscript and the Supplementary Materials.

## Conflict of Interest Statement

The authors declare that the research was conducted in the absence of any commercial or financial relationships that could be construed as a potential conflict of interest.
