# Supplementary material for: Diversity and Dynamics of Microbial Community Structure in Different Mangrove, Marine and Freshwater Sediments During Anaerobic Debromination of PBDEs
Source: Front Microbiol. 2018 May 15;9:952. doi: 10.3389/fmicb.2018.00952 (PMC5962692; doi:10.3389/fmicb.2018.00952)

Supplementary material

Diversity and dynamics of microbial community structure in different mangrove, marine and freshwater sediments during anaerobic debromination of PBDEs

Yafen Wang[1,2,*], Haowen Zhu[2], Ying Wang[2], and Nora Fung-yee Tam[2,3]

[1]Laboratory of Basin Hydrology and Wetland Eco-restoration, School of Environmental Studies, China University of Geosciences, 430074Wuhan, Hubei, PR China
[2]Department of Biology and Chemistry, City University of Hong Kong, Tat Chee Avenue, Kowloon, Hong Kong SAR
[3]State Key Laboratory in Marine Pollution, City University of Hong Kong, Tat Chee Avenue, Kowloon, Hong Kong SAR

*Corresponding author:
Dr. Yafen Wang,
E-mail: wangyf@cug.edu.cn

School of Environmental Studies,
China University of Geosciences,
No. 388 Lumo Road, Hongshan District,
Wuhan 430074, Hubei, PR China

Supplementary Figure S1 Geographical locations of the five mangrove swamps (red circle; STK: Sha Tau Kok, TK: Ting Kok; HC: Ho Chung; MP: Mai Po; TO: Tai O), two freshwater ponds (green circle; NSW: Nam Sang Wai; MPf: Mai Po freshwater) and one marine sediment (blue circle; SK: Sai Kung).

Supplementary Figure S2 Cluster analysis of the eight 16S rRNA gene clone libraries at phylum levels with Ward's method. Site abbreviations refer to Figure 4.

Supplementary Figure S3 Relative abundances of *Dehalococcoides* 16S rRNA genes in the eight sediment microcosms from Day 1 to Day 90.

Supplementary Figure S1

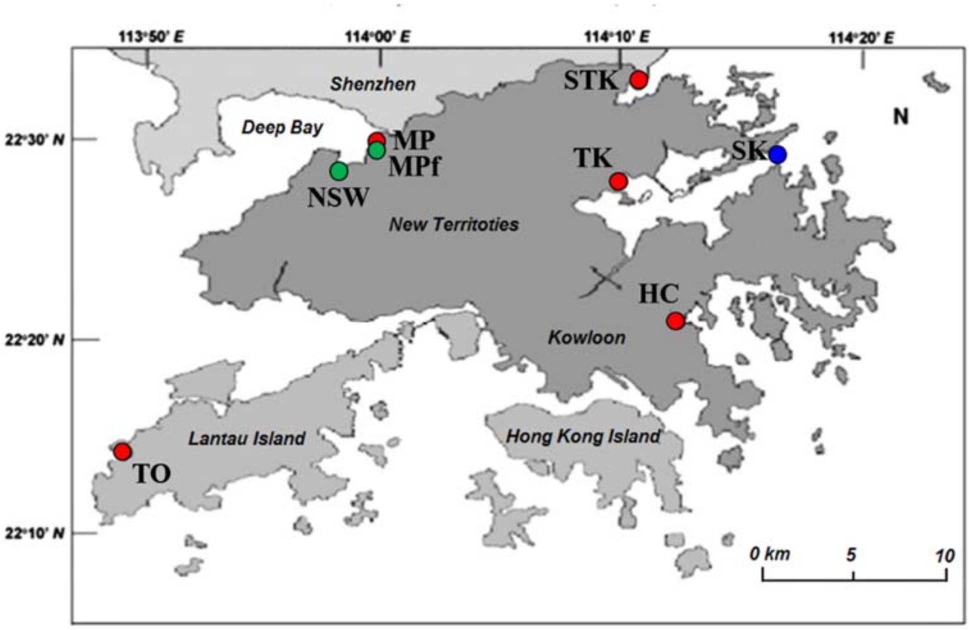

Supplementary Figure S2

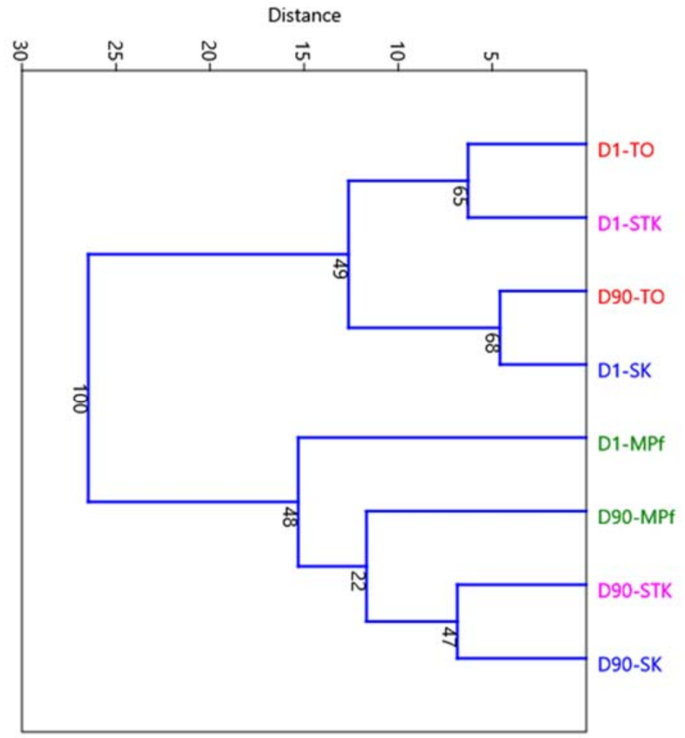

Supplementary Figure S3

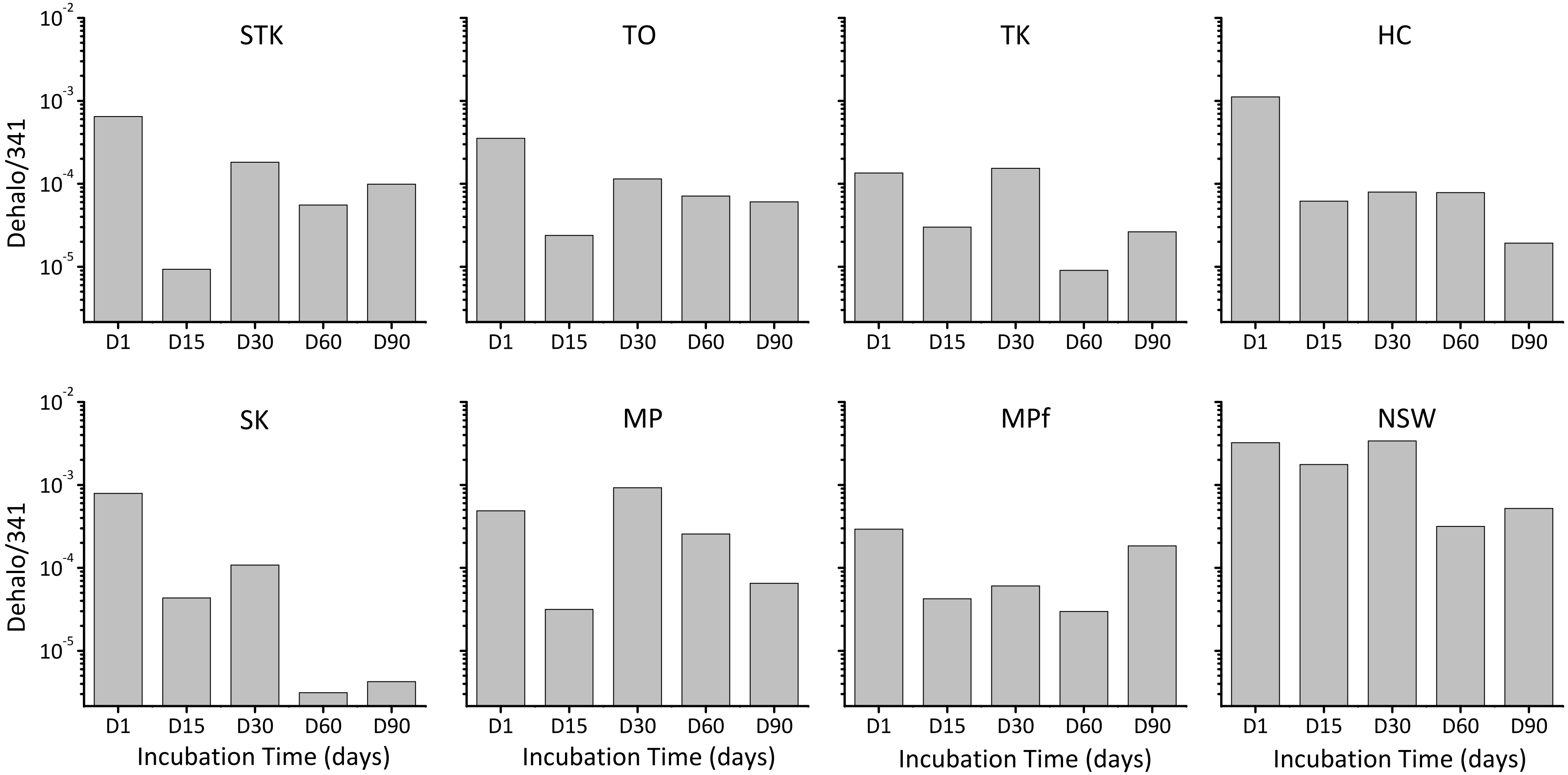

Supplement: Supplementary file 1 [file Image_1.pdf]
